# Targeted-Capture Next-Generation Sequencing in Diagnosis Approach of Pediatric Cholestasis

**DOI:** 10.3390/diagnostics12051169

**Published:** 2022-05-07

**Authors:** Marion Almes, Anne Spraul, Mathias Ruiz, Muriel Girard, Bertrand Roquelaure, Nolwenn Laborde, Fréderic Gottrand, Anne Turquet, Thierry Lamireau, Alain Dabadie, Marjorie Bonneton, Alice Thebaut, Babara Rohmer, Florence Lacaille, Pierre Broué, Alexandre Fabre, Karine Mention-Mulliez, Jérôme Bouligand, Emmanuel Jacquemin, Emmanuel Gonzales

**Affiliations:** 1 Pediatric Hepatology and Pediatric Liver Transplant Unit, Reference Center for Biliary Atresia and Genetic Cholestasis, FSMR FILFOIE, ERN RARE LIVER, Bicêtre Hospital, Assistance Publique-Hôpitaux de Paris (AP-HP), Paris-Saclay University, 94270 Le Kremlin-Bicêtre, France; alice.thebaut@aphp.fr (A.T.); emmanuel.jacquemin@aphp.fr (E.J.); emmanuel.gonzales@aphp.fr (E.G.); 2 INSERM UMR-S 1193, Paris-Saclay University, Hépatinov, 91400 Orsay, France; anne.spraul@aphp.fr; 3 Plateforme d’Expertise Maladies Rares Paris-Saclay, AP-HP, 94270 Le Kremlin-Bicêtre, France; jerome.bouligand@aphp.fr; 4 Biochemistry Unit, DMU15, Bicêtre Hospital, AP-HP, Paris-Saclay University, 94270 Le Kremlin-Bicêtre, France; 5 Pediatric Gastroenterology, Hepatology and Nutrition Unit, Bron Hospital, 69677 Lyon, France; mathias.ruiz@chu-lyon.fr (M.R.); barbara.rohmer@chu-lyon.fr (B.R.); 6 Pediatric Gastroenterology, Hepatology and Nutrition Unit, Necker Hospital, 75015 Paris, France; muriel.girard@aphp.fr (M.G.); florence.lacaille@aphp.fr (F.L.); 7 Pediatric Gastroenterology, Hepatology and Nutrition Unit, Marseille University Hospital, 13288 Marseille, France; bertrand.roquelaure@ap-hm.fr (B.R.); alexandre.fabre@ap-hm.fr (A.F.); 8 Pediatric Gastroenterology, Hepatology and Nutrition Unit, Toulouse Hospital, 31300 Toulouse, France; laborde.n@chu-toulouse.fr (N.L.); broue.p@chu-toulouse.fr (P.B.); 9 Department of Pediatric Gastroenterology Hepatology and Nutrition, Univ. Lille, CHU Lille, INSERM U1286, 59000 Lille, France; frederic.gottrand@chru-lille.fr (F.G.); karine.mulliez@chru-lille.fr (K.M.-M.); 10 Pediatric Gastroenterology, Hepatology and Nutrition Unit, Saint-Denis Hospital, 97405 La Réunion, France; anne.turquet@chu-reunion.fr; 11 Pediatric Gastroenterology, Hepatology and Nutrition Unit, Bordeaux Hospital, 33076 Bordeaux, France; thierry.lamireau@chu-bordeaux.fr; 12 Pediatric Gastroenterology, Hepatology and Nutrition Unit, Rennes Hospital, 35033 Rennes, France; alain.dabadie@chu-rennes.fr; 13 Pediatric Gastroenterology, Hepatology and Nutrition Unit, Nancy Hospital, 54035 Nancy, France; m.bonneton@chru-nancy.fr; 14 Molecular Genetics, Pharmacology and Hormonology Unit, Bicêtre Hospital, AP-HP, Paris-Saclay University, 94270 Le Kremlin-Bicêtre, France; 15 INSERM UMR 1185, Endocrine Physiology and Physiopathology, Paris-Saclay University, 94270 Le Kremlin-Bicêtre, France

**Keywords:** genetic cholestasis, children, NGS, neonatal sclerosing cholangitis, PFIC, Alagille syndrome, transient neonatal cholestasis

## Abstract

Background: Cholestasis is a frequent and severe condition during childhood. Genetic cholestatic diseases represent up to 25% of pediatric cholestasis. Molecular analysis by targeted-capture next generation sequencing (NGS) has recently emerged as an efficient diagnostic tool. The objective of this study is to evaluate the use of NGS in children with cholestasis. Methods: Children presenting cholestasis were included between 2015 and 2020. Molecular sequencing was performed by targeted capture of a panel of 34 genes involved in cholestasis and jaundice. Patients were classified into three categories: certain diagnosis; suggested diagnosis (when genotype was consistent with phenotype for conditions without any available OMIM or ORPHANET-number); uncertain diagnosis (when clinical and para-clinical findings were not consistent enough with molecular findings). Results: A certain diagnosis was established in 169 patients among the 602 included (28.1%). Molecular studies led to a suggested diagnosis in 40 patients (6.6%) and to an uncertain diagnosis in 21 patients (3.5%). In 372 children (61.7%), no molecular defect was identified. Conclusions: NGS is a useful diagnostic tool in pediatric cholestasis, providing a certain diagnosis in 28.1% of the patients included in this study. In the remaining patients, especially those with variants of uncertain significance, the imputability of the variants requires further investigations.

## 1. Introduction

In children, cholestatic jaundice is a rare condition occurring with an estimated prevalence of 1/2500 term infants [1,2]. In older children, the prevalence is certainly lower but is not known. Several etiologies must be investigated in cholestatic children, depending on the patient’s age. Genetic cholestatic diseases collectively represent up to 25% of these etiologies, and might prove difficult to distinguish one from another [2,3].

Many genes related to cholestatic diseases have been identified and continue to be reported, including those responsible for the different progressive familial intrahepatic cholestasis (PFIC), benign recurrent intrahepatic cholestasis (BRIC), Alagille syndrome (AGS), neonatal sclerosing cholangitis (NSC), or inborn errors of bile acids metabolism. Molecular analysis by targeted-capture next generation sequencing (NGS) has recently emerged as a powerful diagnostic tool, allowing sequencing of a panel of selected genes in a single exam [4,5] and reducing both the delay and the cost of the diagnosis. It can be used as the first-line molecular technique or when previous Sanger sequencing of one or more genes failed to identify a precise diagnosis [6].

The main objective of our study was to evaluate from a clinical point of view the performance of NGS using a gene panel dedicated to cholestasis in a large cohort of children with cholestasis. The secondary objective is to report the clinical and paraclinical presentation of patients presenting an unexpected phenotype with respect to genotype.

## 2. Patients and Methods

### 2.1. Type of Study

We conducted a retrospective, observational cohort study. Recruitment was multicentric (Besançon, Béziers, Bordeaux, Clermont-Ferrand, Créteil, Dijon, Lille, Lyon, Nancy, Nice, Orléans, Marseille, Mayotte, Montpellier, Paris-Bicêtre, Paris-Necker, Paris-Trousseau, La Réunion, Nice, Saint-Étienne, Rouen, Rennes, Reims, Strasbourg, Toulouse). All molecular studies were performed in Bicêtre hospital and analyzed by the same biologists (AS and JB).

### 2.2. Patients

We included all patients under 18 years old who underwent a NGS using a gene panel dedicated to cholestasis as part of the diagnostic workup of a cholestatic condition (chronic or transient), between January 2015 and October 2020. Patients whose primary indication for a NGS molecular study was not cholestasis (e.g., acute liver failure and vascular liver disorders) were excluded from the study, as were patients presenting with cholestasis due to biliary atresia.

Our population mostly included patients who were studied prospectively, as part of their initial cholestasis assessment. A minority of patients were included retrospectively after Sanger sequencing of one or more genes if no diagnosis was provided by this technique.

Cholestasis was defined by an increase of serum conjugated bilirubin above 17 µmol/L (1 mg/dL) as recommended [2], and/or an increase of serum bile acids above 15 µmol/L.

### 2.3. Ethics

This study complies with the General Data Protection Regulation (RGPD—Regulation (EU) No. 2016/679) and the French Data Protection Act. The research has been declared and listed in the Data Processing Registry of the Assistance Publique-Hôpitaux de Paris under No. 20191112110112.

The child’s referring physician obtained written informed consent of the patient’s parents, in accordance with the local guidelines established by the hospital’s ethics committee.

### 2.4. Data Collection

When available, the following data as part of the diagnosis work up and of the follow-up were collected from patients’ paper or computerized medical records.

Demographic data included date of birth, sex, age at onset of cholestasis, and age at the time of sampling. Clinical data included prematurity, perinatal anoxo-ischemia, infections, or suspected early-onset neonatal bacterial infections requiring antibiotics, intrauterine growth restriction (IUGR), stool color, hepatomegaly, splenomegaly, and abnormal extra-hepatological features. Biological parameters collected included: liver tests (aspartate amino transferase (AST), alanine amino transferase (ALT), serum gamma-glutamyl transferase (GGT) activity, total and conjugated bilirubin, serum bile acid (sBA) levels), coagulation tests (clotting factor V, prothrombin time (PT) and alpha-fetoprotein.

Imaging studies include mainly abdominal ultrasound (US) and in some patients MRI-cholangiography or cholecystography. Histological analyses of liver biopsy (LB) specimens were recorded.

### 2.5. Clinical Presentation Categories

We defined four categories of clinical presentation, based on the latest clinical and paraclinical (biological, radiological, histological) data available: chronic cholestasis (CC), transient neonatal cholestasis (TNC), benign recurrent intrahepatic cholestasis (BRIC) and oestroprogrestative-induced cholestasis (CholOP). CC was considered if the patient presented chronic liver disease, based on abnormal liver function tests (LFT) or abnormal abdominal imaging persisting after at least one year of treatment with ursodeoxycholic acid (UDCA). TNC definition criteria were: (1) normalization of LFT, with or without UDCA treatment, before the age of one year, and (2) persisting normal liver tests and imaging assessed after the age of one year and at least (3 months after discontinuation of UDCA treatment. BRIC was defined as recurrent episodes of cholestasis, with normalization of LFT between episodes. CholOP was defined as oestroprogestative-induced episodes of cholestasis, resolutive after treatment discontinuation.

### 2.6. Sequencing Technique

#### 2.6.1. Panel Design

A set of genes known to be involved or likely involved in various pediatric liver diseases, including 34 genes involved in cholestasis and jaundice, was selected to form the gene panel. Table 1 lists the 34 genes of the panel known to be involved in genetic cholestasis or jaundice.

The design of this panel was realized via the Agilent design platform (Agilent, Santa Clara, CA, USA). We adjusted the gene panel 4 times during this work, based on the data available in the literature. In this work, we report the results of versions 1 to 4 of the panel (Version 1: from January 2015 to November 2016, Version 2: from December 2016 to February 2018, Version 3: from March to April 2019, Version 4: from May 2019 to October 2020). The different versions of the panel used during this work are presented in Figure S1.

#### 2.6.2. Technical Validation of the Targeted Gene Panel NGS

Thirty patients with CC, in whom polymorphisms in one of the genes included in the panel (*ABCB4*, *ABCB11*, *ATP8B1*, *JAG1*, *NOTCH2*, *ATP7B*) had been previously identified by Sanger sequencing, were used as controls to validate the NGS technique. These patients were not included in the rest of the study.

#### 2.6.3. DNA Extraction

Patients’ DNA was extracted from circulating leukocytes previously obtained from peripheral blood, and purified using a QiaSymphonyMidiKit^®^ kit. It was then fragmented by sonication using a Covaris M220 kit (Covaris Inc., MS, Woburn, MA, USA).

#### 2.6.4. Libraries Constitution

The Illumina^®^ libraries of the entire genome were prepared using the NEBNext^®^ DNA Library Prep preparation kit for Illumina^®^ (NEB Inc., Ipswich, MA, USA) on a Biomek Span 8 workstation (Beckman, Villepinte, France). Enrichment was achieved by targeted capture of the coding exons of the panel genes and their intronic bases (+/−30 base pairs) by hybridization, using the SureselectXT kit (Agilent, Santa Clara, CA, USA) robotized on a Biomek 4000 substation (Beckman, Villepinte, France). Deep intronic regions were not covered by this analysis. Some poorly covered exons were verified in Sanger sequencing if the clinical suspicion was high.

#### 2.6.5. Sequencing Device

Sequencing was performed on a MiSeq Illumina^®^ medium flow sequencer.

### 2.7. Bioinformatics Analysis

#### 2.7.1. Selection of Variants of Interest

Sequencing was considered for further analyses when a coverage superior to 20X was obtained in more than 95% of the targeted regions.

The first selection of non-single nucleotide variants (SNV) mutations was carried out automatically by the Galaxy^®^ Bioinformatics Platform of Paris-Sud University. In a simplified way, the readings of the Fatsq end pairs were taken after alignment of the sequences with the reference human genome 19 (hg19) using the BWA-MEM 0.7.10 software. Variant selection was made using GATK 3.4–46 and SNV variants were annotated using Annovar software (version 2015) and Snpeff 4.0. This first selection allowed, for each gene, the extraction of variants whose allele frequency in the general population was less than 2% except for *CFTR* for which an allele frequency cut off of 5% was used. Copy number variations were analyzed using an in-house Python script that compared depth and coverage data generated by Picard Metrics, as previously reported [7].

A second phase of analysis was then conducted by a specialized biologist who took a specific look at the variants presented in the genes of interest, according to the clinical presentation of the patient, as described below.

#### 2.7.2. Classification of Variants

All mutations are reported following Human Genome Variation Society nomenclature (http://varnomen.hgvs.org/, accessed on 14 March 2022).

The different variants were classified according to the American College of Medical Genetics (ACMG) classification: pathogenic (P, class 5), likely pathogenic (LP, class 4), undetermined significance (VOUS, class 3), likely benign (class 2), and benign (class 1) [8].

All variants, except CFTR variants, were studied through the VarSome^®^ platform (, accessed on 14 March 2022), which provided a class of pathogenicity, according to the ACMG classification. The classification proposed by the platform was generally accepted, unless data supporting the pathogenicity of a variant was available in the literature but not included in the VarSome^®^ database, or if the pathogenicity of a variant was supported by familial studies.

*CFTR* variants were classified according to the recommendations of the National Association of Practitioners of Molecular Genetics (ANPGM) and using the CFTR-France website database (https://cftr.iurc.montp.inserm.fr/cftr, accessed on 14 March 2022). They were sorted into 4 classes [9]: cystic fibrosis-causing (CF Causing), CFTR-related diseases causing (CFTR-RD causing), and variants of undetermined significance (VOUS) for CF or for CFTR-RD mutations.

### 2.8. Variants Reported

All P and LP variants were reported. VOUS were not reported unless they were relevant when confronted to phenotype and their pathogenicity was supported by literature or in silico studies (VOUS-likely pathogenic, VOUS-LP). Benign and likely benign variants were not reported.

When possible, allelic repartition was studied. Copy number variations detected by the NGS technique were confirmed using quantitative PCR, MLPA, or CGHarray.

### 2.9. Diagnostic Categories

Molecular data were compared with clinical and paraclinical findings, allowing to classify the patients into one of the following diagnostic categories.

Provided that molecular data were consistent with phenotypes, patients with biallelic P/LP gene variants for autosomal recessive diseases or P/LP variants for autosomal dominant diseases were classified as having a certain diagnosis for conditions with an available OMIM or ORPHANET-number, or as having a suggested diagnosis for conditions without any available OMIM or ORPHANET-number, namely transient neonatal cholestasis, oestroprogestative-induced cholestasis and chronic or recurrent cholestasis due to monoallelic *ABCB4* deficiency.

Patients were classified as having an uncertain diagnosis when phenotype was not in accordance with the one expected given the molecular findings, or when the molecular findings did not allow the patient to be classified as having a certain or suggested diagnosis (because allelic repartition was not studied and/or because the variant identified was a VOUS).

When the NGS study did not identify any P or LP variants, patients were classified as having no diagnosis.

## 3. Results

### 3.1. Patients

Among the 706 children who were studied through the NGS panel between January 2015 and October 2020, 104 were excluded because they presented biliary atresia or primary non-cholestatic liver disease.

Among the 602 patients included, 309 (51.3%) were under six months old; 357 (59.3%) were male. Two hundred patients were recruited directly by our center (33.2%), while the rest of them were sampled in their local reference center and referred only for genetic testing.

Among the 602 patients included, 65 had been previously studied by Sanger Sequencing.

### 3.2. Technical Validation Phase of Gene Panel

All the polymorphisms identified by Sanger sequencing in the 30 control patients were also identified by targeted NGS.

### 3.3. Depth and Coverage of Sequenced Regions

The average reading depth of the targeted regions was 136X (SD 20.6). Coverage greater than 20X was obtained for more than 98% of the targeted regions.

### 3.4. Patients with a Certain Diagnosis

Targeted gene panel NGS established a certain diagnosis of OMIM/ORPHANET-identified diseases in 169 out of the 602 patients (28.1%), including 24 patients who had previously been studied by Sanger Sequencing (Table 2). All of them had an initial clinical presentation, a biological and clinical evolution consistent with the results of NGS sequencing.

Among the 92 patients who presented with high GGT activity cholestasis, 55 were diagnosed with Alagille syndrome, bearing either a *JAG1* mutation (*n* = 48) or a *NOTCH2* mutation (*n* = 7), 17 patients were diagnosed with PFIC3, nine with neonatal sclerosing cholangitis related to *DCDC2* deficiency, three with cystic fibrosis, three with Wilson disease and five with alpha one anti-trypsin (A1AT) deficiency.

Among the 57 patients who presented with low or GGT normal activity two were diagnosed with FIC1 deficiency (PFIC1, *n* = 1; BRIC, *n* = 1), 22 were diagnosed with BSEP deficiency (PFIC2, *n* = 19; BRIC2, *n* = 3), eight patients were diagnosed with TJP2 deficiency (PFIC4), 10 patients were diagnosed with isolated cholestasis related to *MYO5B* deficiency, five patients with an arthrogryposis—renal failure—cholestasis syndrome (ARC syndrome, related to *VPS33B* deficiency), primary bile acid synthesis defects due to acyl-CoA oxidase deficiency was diagnosed in two patients (*ACOX2*), and spinocerebellar ataxia was diagnosed in three patients (*SCYL1*). The following diseases were all diagnosed in a single patient: NR1H4 deficiency (PFIC5), primary bile acid synthesis deficiency due to delta 4-3-oxoid dehydrogenase deficiency (*AKR1D1*), primary bile acid synthesis deficiency due to bile acid conjugation defects (*BAAT*), cerebrotendinous xanthomatosis (*CYP27A1*) and tricho-hepato-enteric syndrome (*TTC37* gene).

In addition, 20 patients were diagnosed with a genetic hyperbilirubinemia syndrome: Dubin-Johnson syndrome (*ABCC2*, *n* = 17), Rotor syndrome (*SLCO1B1/B3*, *n* = 1), Crigler-Najjar syndrome (*UGT1A1*, *n* = 1) and Gilbert syndrome (*UGTA1*, *n* = 1). Of note, all patients diagnosed with Dubin-Johnson syndrome presented with TNC.

Table S1 in Supplementary Materials reports all causal mutations identified in these patients, as well as other pathogenic variants found in other genes of interest.

### 3.5. Patients with a Suggested Diagnosis

A suggested diagnosis was proposed for 40 patients (6.6%), for whom molecular studies were consistent with known cholestatic conditions without any available OMIM/ORPHANET-number.

A TNC related to pathogenic or likely pathogenic monoallelic mutations in the main PFIC genes was identified in 22 patients (*ATP8B1*, *n* = 2; *ABCB11*, *n* = 12; *ABCB4*, *n* = 8) (Table 3). Importantly, usual risk factors for TNC were disclosed in only 5 of these 22 patients (neonatal CMV infection (*n* = 4) and mild perinatal anoxo-ischemia (*n* = 1)). Patients presenting TNC but in whom no mutation was found were not reported.

Fourteen patients presented with chronic or recurrent high GGT level cholestasis and one PV or LPV in *ABCB4* (Table 3b). Of note, the c.1769G > A variant of *ABCB4* was reclassified as a class 4 variant based on previous studies showing its pathogenic nature [10,11]. This variant was also identified in one compound heterozygous patient of our cohort allowing a certain diagnosis of PFIC3 (patient 187, Table S1 of the Supplementary Materials).

Four patients with oestroprogestative-induced cholestasis associated with one PV or LPV in *ABCB11* are reported in Table 3c (*n* = 4).

**Table 3:** **(a)**. Suggested diagnosis of cholestatic conditions without OMIM/ORPHANET-number: patients with transient neonatal cholestasis due to pathogenic or likely pathogenic heterozygous mutations in one of the main PFIC genes (*n* = 22). **(b).** Suggested diagnosis of cholestatic conditions without OMIM/ORPHANET number: patients with chronic or recurrent cholestasis due to pathogenic or likely pathogenic heterozygous variants in *ABCB4* (*n* = 14). **(c).** Suggested diagnosis of cholestatic conditions without any available OMIM/ORPHANET number: patients with oestroprogestative-induced cholestasis (*n* = 4).

**a**				
**Phenotype**	**Gene**	**Patient**	**Mutation**	**Notes**
Transient neonatal cholestasis	*ATP8B1*	1	c.3040C>T:p.Arg1014Ter	TJP2: p.Gly538Ala (He)—Pathogenic MYO5B: p.Glu144Gly (He) -VOUS
		2	Complete gene deletion	
	*ABCB11*	3	c.3691C>T:p.Arg1231Trp	
		4	c.1445A>G:p.Asp482Gly	
		5	**c.2873_2874insCG:p.Phe959GlyfsTer49**	
		6	c.3329C>T:Ala1110Val	
		7	c.1243C>T:p.Arg415Ter	
		8	**c.3181_3184del:p.Ile1061ValfsTer35**	
		9	**c.2061C>G:p.Tyr687Ter**	Neonatal cholestasis with pale stool and hypoglycemia, normal cholangiogram, advanced fibrosis on LB (at the age of 2 months), normalization of LFT at 1 year old with UDCA treatment. Persistent normal LFT after UDCA discontinuation.
		10	**c.3752C>T:p.Thr1251Ile**	
		11	**c.896-897GA>TT:p.Arg299Ile**	
		12	c.890A>G:p.Glu297Gly	Inherited from his mother who presented ICP—Neonatal CMV infection.
		13	c.3148C>T:p.Arg1050Cys	
		14	c.1396C>A:p.Gln466Lys	Neonatal CMV infection.
	*ABCB4*	15	c.140G>A:p.Arg47Gln	
		16	c.2800G>A:p.Ala934Thr	Neonatal CMV infection.
		17	c.2800G>A:p.Ala934Thr	
		18	c.2800G>A:p.Ala934Thr	
		19	c.101C>T:p.Thr34Met	
		20	c.1769G>A:p.Arg590Gln * [10,11]	Mild perinatal anoxo-ischemia.
		21	**c.3280-2A>G:p(?)**	
		22	**c.3676T>G:p.Cys1226Gly**	
**b**				
**Phenotype**	**Patient**	**Mutation**	**Status**	**Notes**
Chronic or recurrent high GGT level cholestasis	23	***ABCB4:* c.1436C>T:p.Pro479Leu**	He: Pathogenic	Recurent episodes of cholestasis and pruritus. Normalization of LFT with UDCA treatment.
	24	*ABCB4:* c.1769G>A:p.Arg590Gln *	He: Likely Pathogenic [10,11]	Anicteric cholestasis associtaed with abdominal pain. Normal abdominal US images, especially no lithiasis. Normalization of LFT with UDCA treatment.
	25	***ABCB4:*c.1714C>T:p.Gln572Ter** *(inherited from the father)*	He: Pathogenic	Chronic cholestasis and pruritus. Hydrocholecystis with possible compression of extrahepatic bile ducts. Family history of liver disease (father: LPAC syndrome).
	26	*ABCB4:*c.1769G>A:p.Arg590Gln^*^	He: Likely Pathogenic [10,11]	Trisomy 21, neonatal cholestasis with intra vesicular sludge, cirrhosis and ascitis. Complete atrioventricular canal. Duodenal atresia and necrotizing enterocolitis requiring parenteral nutrition. Possible ischemic cholangiopathy due to complicated surgeries during neonatal period. Normalization of LFT at 1 year old, no discontinuation.
	27	*ABCB4:*c.2800G>A:p.Ala934Thr	He: Likely pathogenic	Chronic cholestasis, cirrhosis.
	28	***ABCB4:* c.574G>T:p.Val192Phe** *(inherited from the mother)* ***ABCB4:* c.3655-6C>G:p.(?)** (de novo)	He: Likely pathogenic He: VOUS	Neonatal cholestasis, pale stool, normal cholangiogram, family history of liver disease (mother and maternal aunt: ICP). Partial improvement of LFT with UDCA treatment.
	29	***ABCB4:* c.662T>C:p.Met221Thr** *(inherited from the father)*	He: Likely pathogenic	Fallot’s tetralogy, nephrocalcinosis, persistent abnormal LFT with UDCA treatment, normal cholangiogram, cirrhosis. No information on neonatal period. No family history of liver disease.
	30	*ABCB4:*c.2800G>A:p.Ala934Thr *(inherited from the mother)* ***ATP8B1:*c.2097+1G>A: p.(?)** *(inherited from the father)*	He: Pathogenic He: Likely Pathogenic	Chronic cholestasis, cirrhosis, portal hypertension, aortic arch abnormalities. Family history of liver disease (mother: ICP).
	31	*ABCB4*:c.2144C>T:p.Thr715Ile	He: Likely Pathogenic	Neonatal cholestasis, deceased during neonatal period (multiorgan failure).
	32	*ABCB4:*c.1769G>A:p.Arg590Gln * [10,11]	He: Likely Pathogenic	Mental retardation, butterfly vertebrae. Lost of follow-up.
	33	*ABCB4:*c.1769G>A:p.Arg590Gln * [10,11]	He: Likely Pathogenic	Chronic cholestasis, cirrhosis, liver histology compatible with neonatal sclerosing cholangitis, LT at 2 years old.
	34	*ABCB4:*c.1769G>A:p.Arg590Gln * [10,11] *ABCB11*:c.470A>G:p.Tyr157Cys (*inherited from the mother*)	He: Likely Pathogenic He: VOUS-LP	Extreme prematurity with severe anoxo-ischemia and long term parenteral nutrition. Neonatal cholestasis. Deceased during neonatal period (multiorgan failure). Post mortem LB: panlobular dissecting fibrosis, ductopenia, hepatocellular cholestasis, disseminated intravascular coagulopation. Possible consequences of a submassive destruction of liver parenchyma. Family history of liver disease (mother: ICP; twin brother: TNC (carrying only *ABCB11* variant); healthy older sister (same genotype): asymptomatic vesicular lithiasis).
	35	*ABCB4:*c.959C>T:p.Ser320Phe	He: Likely Pathogenic	Complex cardiac malformation, chronic cholestasis, exsudative enteropathy.
	36	*ABCB4:*c.140G>A:p.Arg47Gln	He: Pathogenic	Patient also diagnosed with a glycogenose storage disease type III and deafness.
**c**				
**Phenotype**	**Gene**	**Patient**	**Mutation**	**Status**
Oestro-progestative induced cholestasis	*ABCB11*	37	c.890A>G:p.Glu297Gly (He) (inherited from her mother) c.403G>A:p.Glu135Lys (He) (de novo or inherited from her father)	CHe
		38	c.150+3A>C:p(?)	He
		39	**c.3130delG:p.Ala1044LeufsTer53**	He
		40	**c.477+6T>G:p(?)**	Ho

ICP: intrahepatic cholestasis of pregnancy, LFT: liver function tests, UDCA: ursodeoxycholic acid. The references cited were used to reclassify the corresponding variant from class 3 to class 4. * The pathogenicity of this variant is supported by previous publications [10,11]. Previously unreported variants according to ClinVar^®^ and Varsome^®^ are indicated in bold. BA: bile acids, ICP: intrahepatic cholestasis of pregnancy, He: heterozygous, LB: liver biopsy, LFT: liver function tests, UDCA: ursodeoxycholic acid, TNC: transient neonatal cholestasis, US: ultrasound. The references cited were used to reclassify the corresponding variant from class 3 to class 4. * The pathogenicity of this variant is supported by previous publications [10,11]. Previously unreported variants according to ClinVar and Varsome are indicated in bold. CHe: compound heterozygous, He: heterozygous, Ho: homozygous. Previously unreported variants according to ClinVar^®^ and Varsome^®^ are indicated in bold.

### 3.6. Patients with an Uncertain Diagnosis

For 21 patients (3.5%), molecular studies led to an uncertain diagnosis (Table 4).

In ten patients, the phenotype was not consistent with the genotype (patients 41 to 50, Table 4). One patient presented with a PFIC3 phenotype, but the variants identified were not fully supportive of this diagnosis and the allelic repartition was not studied (patient 46). One patient presented one PV in *HNF1B* but his clinical presentation was not concordant with data previously reported (patient 44) [12]. Two patients presented a CC with heterozygous PV or LPV in two different genes (patients 47–48, Table 4). In patient 45 (Table 4), NGS revealed a homozygous VOUS variant in *ATP7B*, previously reported to cause an attenuated phenotype of Wilson disease [13]. He presented with CC, cirrhosis, and normal cupric balance but elevated cupric levels in the LB specimen. Patient 49 (Table 4) presented with a PFIC2 phenotype, but only one PV in *ABCB11* was identified.

Eleven patients were carriers for CFTR RD variants, including one patient presenting a homozygous status (Table 4). Among them, four had a transient neonatal cholestasis and seven presented with CC. Of note, no variant (P, LP, VOUS) in the other genes included in the panel was identified in these patients. In addition, no usual risk factors for TNC were disclosed in the four patients with TNC.

**Table 4:** **(a)**. Uncertain diagnosis of cholestatic conditions (*n* = 10). **(b).** Uncertain molecular diagnosis: carriers for CFTR-related disease (RD) or CF-causing mutations (*n* = 11).

**a**			
**Patient**	**Genotype**	**ACMG Classification**	**Phenotype**
41	***NOTCH2*:c.1396C>T:p.Gln466Ter (He)**	Pathogenic	Chronic cholestasis, no extra-hepatic AGS feature, neonatal occlusion, neonatal CMV infection, no bile duct paucity in a LB containing only 6 portal spaces.
42	***NOTCH2*:c.5002+2T>C:p.(?) (He)**	Pathogenic	Transient neonatal cholestasis, No extra-hepatic AGS feature. No LB, no UDCA treatment.
43	***NOTCH2*:c.6101C>A:p.Ala2034Asp (He)**	VOUS-LP	Chronic cholestasis, No extra-hepatic AGS feature, no bile duct paucity on LB.
44	*HNF1B*:1.32Mb deletion including HNF1B (He)	Pathogenic	Acute hepatitis with anicteric cholestasis, spontaneous resolution, no relapse (3 years of follow-up). No diabetes. Normal renal function. Slight perisinusoidal fibrosis, moderate hyperplasia of stellate cells and normal bile ducts in LB. Normal liver and renal ultrasound.
45	*ATP7B:*c.4135C>T:p.Pro1379Ser* (Ho) [13]	VOUS	Chronic cholestasis, cirrhosis, normal cupric balance but elevated cupric levels in LB.
46	*ABCB4:*c.2581T>G:p.Leu861Val (He) *ABCB4:*c.1584G>C:p.Glu528Asp ^¤^ (He) [14]	VOUS-LP VOUS	Chronic cholestasis, cirrhosis on LB (MDR3 staining: not available).
47	***NR1H4:* c.920-2A>G:p.(?) (He)** *DGUOK:* c.353G>A:p.Arg118His (He)	Pathogenic Likely Pathogenic	Neonatal cholestasis with hypoglycemia; steatosis and hepatocellular cholestasis on LB. Treated with UDCA: normal LFT and abdominal US at age 2 years. No discontinuation of UDCA.
48	***MYO5B:* c.3190C>T:p.Arg1064Ter** (He) *(inherited from the father)* *CFTR:* c.3485G>T p.Arg1162Leu (He) *(inherited from the mother)*	Pathogenic RD CAUSING	Pruritus, chronic diarrhea, chronic bronchial congestion, normal cholangiogram.
49	*ABCB11*:c.1408C>T:p.Arg470Ter (He)	Pathogenic	Chronic cholestasis persistent with UDCA treatment, cirrhosis.
50	*ABCB11*:c.890A>G:p.Glu297Gly (He)	Likely Pathogenic	Neonatal cholestasis. Lost of follow-up.
**b**			
**Patient**	**Genotype**	**CFTR-France Classification**	**Phenotype**
51	*CFTR*:[c.2002C>T:p.Arg668Cys c.1727G>C:p.Gly576Ala] (He)	RD CAUSING RD CAUSING	Transient neonatal cholestasis
52	*CFTR*:c.2991G>C:p.Leu997Phe (He)	RD CAUSING	
53	*CFTR*:c.3909C>G:p.Asn1303Lys (He)	CF-causing	
54	*CFTR*:[c.220C>T:p.Arg74Trp; c.3808G>A:p.Asp1270Asn] (Ho)	RD-VOUS4 RD-VOUS4	
55	*CFTR*:[c.220C>T:p.Arg74Trp; c.3808G>A:p.Asp1270Asn; c.601G>A: p.Val201Met] (He)	RD-VOUS4 RD-VOUS4 RD-VOUS4	Chronic cholestasis
56	*CFTR*: c.1523T>G:p. Phe508Cys (He) [c.1727G>C:p.Gly576Ala; c.2002C>T:p.Arg668Cys] (He)	RD CAUSING RD CAUSING RD CAUSING	
57	*CFTR*:c.2991G>C:p.Leu997Phe (He)	RD CAUSING	
58	*CFTR*:c.2991G>C:p.Leu997Phe (He)	RD CAUSING	
59	*CFTR*:c.3485G>T:p.Arg1162Leu (He)	RD CAUSING	
60	*CFTR*:c.2173G>A:p.Glu725Lys (He)	RD CAUSING	
61	*CFTR*: c.1516A>G:p.Ile506Val (He)	RD-VOUS4	

AGS: Alagille syndrome, He: heterozygous, Ho: homozygous, VOUS: variant of uncertain significance, VOUS-LP: variant of uncertain significance-likely pathogenic, RD CAUSING: CFTR-related disease causing, LB: liver biopsy, LFT: liver function tests, UDCA: ursodeoxycholic acid. *, the pathogenicity of this variant is supported by a previous publication [13], ^¤^ the pathogenicity of this variant is supported by a previous publication [14]. Previously unreported variants according to ClinVar^®^ and Varsome^®^ are indicated in bold. He: heterozygous, VOUS4: VOUS-likely pathogenic (CFTR specific denomination).

### 3.7. Patients with No Diagnosis

372 patients (61.8%) remained undiagnosed after performing NGS sequencing.

## 4. Discussion

Genetic cholestasis encompasses an increasing number of rare and severe diseases. Clinical findings and biological presentation are often similar. An early diagnosis in these patients may offer more specific therapeutics, delay invasive diagnostic procedures (biliary tree opacification, LB), and help clinicians with genetic counseling. Thus, NGS seems to be a first choice tool in the diagnostic process of these diseases [3,15].

NGS sequencing of genes involved—or potentially involved—in pediatric cholestatic diseases in children allowed us to establish a certain diagnosis in 169 patients (28.1%). This diagnostic performance rate was similar to those previously reported in literature (Table 5) [15,16,17,18,19,20]. Two studies reported significantly higher diagnostic performance rates of 61% and 68% [21,22]. However, the first study was performed in a population with a high inbreeding rate [21] while the second one reported on a population mainly made of retrospective patients that had not been previously studied by Sanger sequencing of the classical PFIC genes [22]. Among the 602 patients included in our study, 65 had been previously studied using the Sanger technique for one or more genes, and had remained undiagnosed. NGS sequencing established a diagnosis for 24 of them (36.9%) (*ABCB4*, *n* = 2; *TJP2*, *n* = 6; *JAG1*, *n* = 2; *DCDC2*, *n* = 2; *ABCC2*, *n* = 1; *AKR1D1*, *n* = 1; *ATP7B*, *n* = 1; *CYP27A1*, *n* = 1; *MYO5B*, *n* = 5; *NR1H4*, *n* = 1; *VPS33B*, *n* = 2). If we only consider the 537 patients in whom NGS was used as first-line diagnosis approach, the diagnostic performance rate decreased slightly to 27%.

Among the 169 patients in whom a certain diagnosis was provided by NGS, we reported patients with an atypical phenotype with respect to genotype. One patient was diagnosed with PFIC3 although he was considered as having a NSC since the first year of life, on the basis of a MRI-cholangiography showing an abnormal intrahepatic biliary tree (patient 188, Tables S1 and S2, Figure S2a). Of note, no variant in the genes involved in NSC was identified in this patient. Interestingly, in our experience, abnormal biliary imaging (transhepatic cholecystography) was observed in one additional PFIC3 patient (not included in this study but detailed in Table S2 and Figure S2b. One hypothesis to explain this abnormal aspect of the biliary tree mimicking NSC, is that MDR3 deficiency reduces phospholipid levels in patient bile, resulting in toxic bile that leads to biliary injuries as has been observed in MDR2−/− mice [23,24]. Such secondary sclerosing cholangitis has not been reported so far in the PFIC3 series [25,26] but has been described in LPAC patients with MDR3 deficiency [27,28].

One patient was diagnosed as a teenager with ARC syndrome (patient 124, Table S1), while previously considered as having an idiopathic CC, associated with tubulopathy and benign osseous abnormalities (talus foot, congenital luxation of both hips). He underwent a LT at four years old before the diagnosis of ARC syndrome was made. Renal dysfunction worsened after LT. Additional explorations, performed after the molecular diagnosis was made, showed perception deafness, frontal syndrome, and thrombopathy (agranular platelets). He died at age 30 of chronic respiratory failure, probably due to long-term medication toxicity (mycophenolate mofetil). Such hypomorphic forms of ARC syndrome have been previously reported [29].

Interestingly, in one patient (patient 82, Table S1) presenting with a clinical diagnosis of AGS, targeted gene panel NGS identified not only a PV in *JAG1* but also one VOUS-LP and one PV in *MYO5B*. While the allelic repartition was not studied, MYO5B immunostaining showed an abnormal pattern consistent with a diagnosis of MYO5B deficiency associated with a *JAG1*-related AGS (unpublished personal data, E. Gonzales). In another patient (patient 218, Table S1), NGS revealed a compound heterozygous status for pathogenic variants both in *MYO5B* and *ATP8B1*. Myo5B deficiency and PFIC1 are both responsible for low GGT chronic cholestasis and possible gastrointestinal manifestations, it is difficult to determine the contribution of each molecular defect to the global phenotype of this patient. No Myo5B immunostaining had been performed in this patient.

Forty patients (6.6%) of the study were classified as having a suggested diagnosis based on a consistent genotype with respect to a phenotype that is not OMIM/ORHANET labeled. In adults, it has been reported that monoallelic PV or LPV variant in *ABCB4* can be associated with CC and cirrhosis consistent with an attenuated phenotype of PFIC3 [10,25]. Interestingly, in this study, we report on 14 children presenting with chronic or recurrent cholestasis with such genotype. Phenotypes ranged from recurrent episodes of high GGT cholestasis to high GGT chronic cholestasis leading to cirrhosis. These observations are in line with those of a recent study and suggest that monoallelic P or LP *ABCB4* variants can also be responsible for chronic cholestatic disease in children [30,31]. We also reported on four female teenagers who presented a first episode of normal GGT cholestasis when started with an oestroprogestative drug and in whom a mono-allelic P or LP variant of *ABCB11* was identified. Such oestroprogestative induced cholestasis has been reported in female patients carrying a monoallelic variant in *ABCB4* [25] and *ABCB11* [32]. These reports highlight the need for specialized gynecological management in collaboration with hepatologists not only in female patients diagnosed with a PFIC but also in female patients known to carry a P or an LP variant in the genes involved in genetic cholestasis especially *ABCB11* and *ABCB4*. Reciprocally, these data suggest that molecular analysis of the genes involved in cholestasis should be considered in female patients presenting a bout of cholestasis after having been started with an oestroprogestative drug. While most of the published cohorts included patients with severe and/or persistent cholestasis [16,17,19,21], our study also included patients presenting with a TNC. Many non-genetic risk factors of TNC have been reported [33,34], and few studies have suggested that monoallelic pathogenic variants in genes involved in genetic cholestasis could contribute to TNC physiopathology [35,36,37,38,39]. Here we report 22 patients presenting with a TNC in whom one monoallelic pathogenic variant of *ABCB11* (*n* = 12), *ABCB4* (*n* = 8), *ATP8B1* (*n* = 2) was identified. Of note, 17 out of these 22 patients did not present any identifiable non-genetic risk factors for CNT. These findings further suggest the role of genetic variants in the pathophysiology of CNT. Whether or not the TNC features of these patients are different from those without genetic risk factors of TNC remains to be studied. In any case, such patients should be discussed with tertiary care center as they may benefit from specific treatment (e.g., prolonged treatment with UDCA) and follow-up (e.g., during pregnancy or when considering oestroprogestative therapy, the possible occurrence of biliary lithiasis and even slow progression fibrosis, as discussed above).

Among the 21 patients (3.5%) classified as having an uncertain diagnosis, we identified a combination of a monoallelic pathogenic variant in two genes in four patients with a CC. One patient (patient 30) presented with a PV in *ABCB4* and a LPV variant in *ATP8B1*. The two proteins encoded by these genes, MDR3 and FIC1 respectively, had been shown to work synergistically to maintain the lipid composition of the canalicular membranes of hepatocytes. Another patient (patient 48) presented with a PV in *MYO5B*, involved in the intracellular traffic of proteins, and a CFTR-RD variant. We cannot exclude that the combination of these variants could explain CC in these patients. NGS sequencing revealed cystic fibrosis in three patients and detected heterozygous *CFTR* variants in 11 patients (ten CFTR-RD variants, one CF causing variant). Four of them had a CC phenotype and seven a TNC phenotype. For these variants, we referred to data available in CFTR specialized databases. Our work raises the hypothesis of a specific cholestasis entity associated with mutations in *CFTR*, different from chronic liver disease associated with CF. This clinical entity could be explained through the dysfunction of the CFTR protein (encoding a chloride channel), leading to disturbances in the hydro-electrolytic balance of the bile and, consequently, to an alteration of bile flow. This hypothesis has already been mentioned in animal models (ICP, in mice [40,41]). Interestingly, patients presenting neonatal cholestasis and a *CFTR* variant have been reported by two other teams [15,38]. Both genetic and phenotypic features of this subtype of cholestasis related to CFTR-RD variants appear to be different from the classical chronic liver diseases associated with CF. Regarding clinical features, data in the literature indicate that CF-associated liver disease usually becomes clinically patent in school-age children and manifests as cirrhosis and portal hypertension rather than as CC. We suggest that cholestasis associated with *CFTR*-RD variants described in this work should better be integrated into “CFTR spectrum pathologies not associated with cystic fibrosis”, such as isolated vas deferens agenesis and diffuse bronchiectasis [9]. This hypothesis is supported by the fact that 6 of the above-mentioned variants are CFTR-RD variants (Table 4b). Further clinical and fundamental studies are needed to strengthen our hypothesis.

Our work also presents limitations that deserve to be detailed. First, regarding the NGS technique itself, detection of mutations involving large variations in CNV (large insertion or deletion mutations) requires a very careful analysis of the databases generated by sequencing, especially in poorly covered regions. qPCR, MLPA, or CGHarray may be required when CNV is suspected in front of abnormal deep coverage ratio (<0.5 or >1.6) in targeted regions. The biologist’s viewpoint in selecting variants of interest in the second phase of bio analysis is very important. This selection is operator dependent: we considered it reproducible in our work because the same person had carried it out for all patients. In addition, mutations in non-coding sequences (deep intronic, or in promoter regions) can also influence the phenotype without being detected by the NGS technique. Moreover, since the number of genes involved in neonatal and infantile cholestasis is constantly evolving, the gene panel must be frequently updated with the addition of new genes. Among the 372 patients remaining undiagnosed in this study (61.8%), those with chronic cholestasis and progressive liver disease are candidates for further molecular analyses, such as last update versions of the gene panel, exome analysis (Whole Exome Sequencing—WES), and genome analysis (Whole Genome Sequencing—WGS). In the setting of a French initiative for genomic medicine aiming to change methods for diagnosis, prevention, and treatment of patients (“Plan France médecine génomique 2025”), these patients with severe liver diseases of pediatric onset can benefit from a WGS (trio analysis). However, it is to be reminded that these studies generate require important bioinformatics analysis, and are more expensive and time-consuming than the analysis of data provided by a panel of targeted genes. Finally, as in all genetic diseases, environmental or epigenetic factors may be involved and must be investigated [42].

## 5. Conclusions

Herein we report the results of more than five years of experience with NGS using a targeted gene panel in the diagnostic work up of children presenting with cholestasis. Used as a first-line diagnostic tool, it provides an etiological diagnosis in 28.1% of patients and completes the routine etiological assessment. The hypothesis of multigenic contribution and imputability of VOUS in patients’ phenotypes require further investigations, both in clinical data collection and functional aspects. Cellular and animal models could contribute to a better understanding of these data. Thus, for some patients, the interpretation of molecular findings should be considered an ongoing process, which deserves to be updated taking into account newly published data and the clinical evolution of the patients.

## Figures and Tables

**Table 1:** Genes of the panel involved in genetic cholestasis or jaundice.

*ABCB11* (NM_003742.2)	*ABCB4* (NM_000443.3)	*ABCC2* (NM_000392.3)	*ACOX2*(NM_003500) *	*AIRE* (NM_000383)
*AKR1D1* (NM_005989.3)	*AMACR* (NM_014324.5)	*ATP7B* (NM_000053)	*ATP8B1* (NM_005603.4)	*BAAT* (NM_001701.3)
*CFTR* (NM_000492.3)	*CIRH1A* (NM_032830.2)	*CLDN1* (NM_021101.4)	*CYP27A1* (NM_000784.3)	*CYP7B1* (NM_004820.3)
*DCDC2* (NM_016356) *	*GBE1* (NM_000158)	*GPBAR1* (NM_001077191)	*HSD3B7* (NM_025193.3)	*JAG1* (NM_000214.2)
*MYO5B* (NM_001080467)	*NOTCH2* (NM_024408.3)	*NR1H4* (NM_00512)	*SERPINA1*(NM_000295) *	*SCYL1* (NM_020680) *
*SLCO1B1/SLCO1B3* (NM_006446/NM_019844)	*SLC25A13* (NM_014251.2)	*SLC27A5* (NM_012254.2)	*TJP2* (NM_004817.3)	*TTC37* (NM_014639.3)
*UGT1A1* (NM_000463.2)	*UNC45A* (NM_018671)	*VIPAS39* (NM_022067.3)	*VPS33B* (NM_018668.3)	

* Only included in some versions of the panel (see detailed versions of each gene panel in Figure S1 of the Supplementary Materials).

**Table 2:** Certain molecular diagnosis of cholestasis or hyperbilirubinemia as determined by the NGS panel (*n* = 170).

Gene	Transmission Mode	Disease (OMIM/Orphanet Number)	Number of Patients
**Cholestasis with High GGT Activity**			**92**
*JAG1*	AD	Alagille syndrome (118450/261619)	48
*NOTCH2*	AD	Alagille syndrome (610205/261629)	7
*ABCB4*	AR	PFIC3 (602347/79305)	17
*DCDC2*	AR	Neonatal sclerosing cholangitis (617394/480556)	9
*CFTR*	AR	Cystic fibrosis (219700/586)	3
*ATP7B*	AR	Wilson disease (277900/905)	3
*SERPINA1*	AR	Alpha-1-antitrypsine deficiency (613490/60)	5
**Cholestasis with Normal or LOW GGT Activity**			**57**
*ATP8B1*	AR AR	PFIC1 (211600/79306) BRIC1 (243300/99960)	1 1
*ABCB11*	AR AR	PFIC2 (601847/79304) BRIC2 (605479/99961)	19 3
*TJP2*	AR	PFIC4 (615878/480483)	8
*NR1H4*	AR	PFIC5 (617049/480476)	1
*MYO5B*	AR	Myosin 5b deficiency related cholestasis (in absence of MVID) (ND/480491)	10
*VPS33B*	AR	ARC syndrome (208085/2697)	5
*AKR1D1*	AR	Primary bile acid synthesis defect (235555/79303)	1
*ACOX2*	AR	Primary bile acid synthesis defect (617308/ND)	2
*BAAT*	AR	Primary bile acid synthesis defect (conjugation defect) (619232/238475)	1
*CYP27A1*	AR	Cerebrotendinous xanthomatosis (213700/909)	1
*SCYL1*	AR	Spinocerebellar ataxia (616719/466794)	3
*TTC37*	AR	Tricho-hepato-enteric syndrome (614602/84064)	1
**Genetic Hyperbilirubinemia**			**20**
*UGT1A1*	AR	Gilbert syndrome (143500/357)	1
	AR	Crigler-Najjar syndrome (218800/79235)	1
*ABCC2*	AR	Dubin-Johnson syndrome (237500/234)	17
*SLCO1B1/B3*	AR	Rotor syndrome (237450/3111)	1

AD: autosomal dominant; AR: autosomal recessive, ARC: Arthrogryposis—renal failure—cholestasis, BRIC: benign recurrent intrahepatic cholestasis, microvillous inclusion disease, ND: not defined; PFIC: progressive familial intrahepatic cholestasis (PFIC).

**Table 5:** Results of NGS sequencing in pediatric patients in diagnosis approach of cholestasis: data already published.

Reference	Number of Genes	Number of Patients	Age (Years)	NGS Indication	Diagnostic Rate (%)
Matte U et al. JPGN. 2010	5	51	0–17	Chronic cholestasis	27%
Wang NL et al. PLoS One. 2016	61	141	0–17	Chronic cholestasis	22%
Togawa T et al. J Pediatr. 2016	18	109	<1	Chronic cholestasis	26%
Stalke A et al. Clin Genet. 2018	21	135	0–20	Chronic cholestasis	17%
Chen HL et al. J. Of Ped. 2018	52	102	0–18	Cholestasis	32,4%
Shagrani et al. Clin Genet. 2018	189	98	0–17	Severe cholestasis	61%
Lipinski et al. Front. Pediatr. 2020	53	22	0–18	Chronic cholestasis	68%
Karpen et al. JPGN. 2021	66	2171	0–18	Cholestasis	12%
**Our study**	**34**	**603**	**0–17**	**Cholestasis**	**28%**

## Supplementary Materials

The following are available online at https://www.mdpi.com/article/10.3390/diagnostics12051169/s1, Figure S1: Details and dates of use of the 4 genes panels, Table S1:Genotype of patients with certain diagnosis (ranged in alphabetic ordrer, regarding the disease, Table S2: Main characteristics of 2 patients with PFIC3 and cholangiopathy mimicking neonatal sclerosing cholangitis, Figure S2. MRI-cholangiography in patient 188 with MDR3 deficiency (PFIC3) and transhepatic cholecystography in an additional PFIC3 patient not included in the study.
