# Supplementary material for: Targeted-Capture Next-Generation Sequencing in Diagnosis Approach of Pediatric Cholestasis
Source: Diagnostics (Basel). 2022 May 7;12(5):1169. doi: 10.3390/diagnostics12051169 (PMC9140938; doi:10.3390/diagnostics12051169)
Supplement: Supplementary file 1 [file diagnostics-12-01169-s001.zip › diagnostics-1660539-supplementary.pdf]

Supplementary Materials

<u>Figure S1</u>: Details and dates of use of the 4 genes panels

Version 1: From January 2015 to November 2016

ABCB11, ABCB4, ABCC2, ABCG5/ABCG8, ACAD9, ACAMD, ACADS, ACADVL, AGL, AIRE, AKR1D1, ALDOB, AMACR, ATOX1, ATP7B, ATP8B1, BAAT, BCS1L, C10orf2, CCBE1, CFC1, CFTR, CIRH1A, CLDN1, CLDN6, CLDN9, COMMD1, CPT1A, CPT1B, CPT2, CYP27A1, CYP7A1, CYP7B1, DGUOK, DLD, ETFA, ETFB, ETFDH, FARS2, FBP1, G6PC, GALT, GBE1, GFM1, GPBAR1, GYG1, GYS1, HADHA, HADHB, HADHSC, HAMP, HFE, HJV, HMGCL, HMGCS2, HSD17B4, HSD3B7, JAG1, KEAP1, KRT18, KRT8, LPIN1, LPIN2, MPV17, MYO5B, NFE2L2, NOTCH2, NR1H4, OCLN, OXCT1, PHKA2, PHKB, PHKG2, PKD1, PKD2, POLG, PYGL, RAB11A, RDX, RFNG, SCO1, SERPINA1, SI, SLC22A5, SLC25A13, SLC25A20, SLC27A5, SLC2A2, SLC2A5, SLC37A4, SLC40A1, SLC5A1, SLCO1B1/SLCO1B3, TALDO1, TFR2, TJP2, TRMU, TUFM, UGT1A1, VIL1, VIPAS39, VPS33B.

Version 2: From December 2016 to February 2018

ABCB11, ABCB4, ABCC2, ABCG5/ABCG8, ABCD3, ADK, AGL, AIRE, AKR1D1, ALDOB, AMACR, AQP8, ATOX1, ATP7B, ATP8B1, BAAT, BCS1L, C10orf2, CCBE1, CFC1, CFTR, CIRH1A, CLDN1, CLDN6, CLDN9, COMMD1, CPT1A, CTC1, CYP27A1, CYP7A1, CYP7B1, DCDC2, DGUOK, DKC1, DLD, EPHX1, FAH, FARS2, FBP1, GAA, GALE, GALT, GBE1, GFM1, GPBAR1, GYG1, GYS2, HMGCL, HMGCS2, HNF1A, HNF1B, HSD17B4, HSD3B7, JAG1, KCNN3, KEAP1, KRT18, KRT8, LFNG, MFNG, MPV17, MYO5B, NBAS, NOTCH1, NOTCH2, NR1H4, OCLN, OXCT1, PHKA2, PHKB, PHKG2, PKHD1, POGLUT1, POLG, PRKCSH, PRSS1, PYGL, RAB11A, RDX, RFNG, RTEL1, SCO1, SEC63, SERAC1, SI, SKIV2L, SLC10A1, SLC10A2, SLC25A13, SLC27A5, SLC2A2, SLC2A5, SLC30A10, SLC4A2, SLC40A1, SLC5A1, SLCO1B1/SLCO1B3, SPINK1, TALDO1, TEM30C, TERC, TERT, TJP2, TRMU, TTC37, TUFM, UGT1A1, VEPH1, VIL1, VIPAS39, VPS33B.

Version 3: From Mars to April 2019

ABCB11, ABCB4, ABCC2, ABCG5/ABCG8, ABCD3, ACOX2, AGL, AIRE, AKR1D1, ALDOB, AMACR, AQP8, ATP7B, ATP8B1, BAAT, CCBE1, CFTR, CIRH1A, CPT1A, CTC1, CYP27A1, CYP7A1, CYP7B1, DCDC2, DGUOK, DKC1, DLD, EPHX1, FAH, FBP1, FOPV, FUT2, GALE, GALT, GATA6, GBE1, GFM1, GPBAR1, GYG1, GYS2, HMGCL, HMGCS2, HSD17B4, HSD3B7, IARS, IFT140, IFT172 (IFTB cpsant), JAG1, KCNN3, LARS, LFNG, MFNG, MPV17, MYO5B, NBAS, NOTCH1, NOTCH2, NR1H4, OXCT1, PHKA2, PHKB, PHKG2, PKHD1, POGLUT1, POLG, PPP1R15B, PRKCSH, PYGL, RAB11A, RDX, RFNG, RTEL1, SCO1, SCYL1, SEC63, SI, SKIV2L, SLC10A1, SLC10A2, SLC25A13, SLC27A5, SLC2A2, SLC2A5, SLC4A2, SLC5A1, SLCO1B1/SLCO1B3, TALDO1, TEM30C, TERC, TERT, TJP2, TRMU, TTC37, TUFM, UGT1A1, UNC45A, VEPH1, VIL1, VIPAS39, VPS33B.

Version 4: From April 2019 to October 2020

ABCB11, ABCB4, ABCC2, ABCG5, ABCG8, ABCD3, ACOX2, AGL, AIRE, AKR1D1, ALDOB, AMACR, AQP8, ATP7B, ATP8B1, BAAT, CCBE1, CFTR, CIRH1A, CPT1A, CTC1, CYP27A1, CYP7A1, CYP7B1, DCDC2, DGUOK, DKC1, DLD, EPHX1, FAH, FBP1, FOPV, FUT2, GALE, GALT, GATA6, GBE1, GFM1, GPBAR1, GYG2, GYS2, HNF1A, HNF1B, HMGCL, HMGCS2, HSD17B4, HSD3B7, IARS, IFT140, IFT172 (IFTB cpsant), JAG1, KCNN3, LARS, LFNG, MARS, MFNG, MPV17, MYO5B, NBAS, NOTCH1, NOTCH2, NR1H4, OXCT1, PHKA2, PHKB, PHKG2, PKHD1, POGLUT1, POLG, PPP1R15B, PRKCSH, PYGL, RAB11A, RDX, RFNG, RTEL1, SCO1, SCYL1, SEC63, SI, SKIV2L, SLC10A1, SLC10A2, SLC25A13, SLC27A5, SLC2A2, SLC2A5, SLC4A2, SLC5A1, SLCO1B1, SLCO1B3, TALDO1, TEM30C, TERC, TERT, THBS2, TKFC, TJP2, TRMU, TTC37, TUFM, UGT1A1, UNC45A, VEPH1, VIL1, VIPAS39, VPS33B.

Table S1. Genotype of patients with certain diagnosis (ranged in alphabetic ordrer, regarding the disease).

Patient	Gene	Status	HGVS	Conclusion	Additional molecular findings
62	*SERPINA1*	Ho	c.1096G>A:p.Glu366Lys	A1AT deficiency	ABCB4: p.Arg590Gln (He)
63	*SERPINA1*	Ho	c.1096G>A:p.Glu366Lys	A1AT deficiency	
64	*SERPINA1*	Ho	c.1096G>A:p.Glu366Lys	A1AT deficiency	ABCB4:p.Thr775Met (He)
65	*SERPINA1*	Ho	c.1096G>A:p.Glu366Lys	A1AT deficiency	
66	*SERPINA1*	Ho	c.1096G>A:p.Glu366Lys	A1AT deficiency	
67	*NOTCH2*	He	c.6007C>T: p.Arg2003Ter	Alagille syndrome	
68	*NOTCH2*	He	**c.5983_5984del:p.Leu1995ValfsTer29**	Alagille syndrome	
69	*NOTCH2*	He	c.4699C>T:p.Arg1567Trp	Alagille syndrome	
70	*NOTCH2*	He	**c.4084_4096del:p.Ser1362AlafsTer61**	Alagille syndrome	
71	*NOTCH2*	He	**c.6806G>T.p.Gly2269Trp**	Alagille syndrome	
72	*NOTCH2*	He	**c.1282G>T:p.Glu428Ter**	Alagille syndrome	ABCB4:p.Arg590Gln (He)
73	*NOTCH2*	He	**c.1276C>T:p.Pro426Ser**	Alagille syndrome	
74	*JAG1*	He	**c.2455A>G:p.Ile819Val**	Alagille syndrome	
75	*JAG1*	He	**c.702C>A:p.Cys234Ter**	Alagille syndrome	
76	*JAG1*	He	c.2122_2125del:p.Gln708ValfsTer34	Alagille syndrome	
77	*JAG1*	He	**c.1304dupA:p.Tyr435Ter**	Alagille syndrome	
78	*JAG1*	He	c.2122_2125del:p.Gln708ValfsTer34	Alagille syndrome	
79	*JAG1*	He	c.2230C>T:p.Arg744Ter	Alagille syndrome	
80	*JAG1*	He	**c.1856_1857del: p.Lys619ArgfsTer9**	Alagille syndrome	
81	*JAG1*	He	**c.2899A>T:p.Lys967Ter**	Alagille syndrome	
82	*JAG1*	He	c.2033delA:p.Asn678MetfsTer65	Alagille syndrome	MYO5B:p.Tyr559Ter (He), p.Leu1216Arg (He)
83	*JAG1*	He	**c.2018_2021dup:p.Asn674fsLysTer16**	Alagille syndrome	
84	*JAG1*	He	c.439C>T:p.Gln147Ter	Alagille syndrome	
85	*JAG1*	He	c.439+1G>A:p.(?)	Alagille syndrome	
86	*JAG1*	He	**c.754del:p.Arg252GlyfsTer160**	Alagille syndrome	
87	*JAG1*	He	**c.1165dupT: p.Cys389LeufsTer6**	Alagille syndrome	
88	*JAG1*	He	**c.3226_3227del:p.Val1076LeufsTer32**	Alagille syndrome	
89	*JAG1*	He	Complete deletion of the gene	Alagille syndrome	Confirmed by MLPA
90	*JAG1*	He	c.1313G>A:p.Cys438Tyr	Alagille syndrome	
91	*JAG1*	He	**c.407_408delinsCT:p(?)**	Alagille syndrome	
92	*JAG1*	He	**c.695-2del:p(?)**	Alagille syndrome	
93	*JAG1*	He	c.delexon16_26:p(?)	Alagille syndrome	Confirmed by MLPA
94	*JAG1*	He	c.488C>G:p.Pro163Arg	Alagille syndrome	
95	*JAG1*	He	c.551G>A:p.Arg184His	Alagille syndrome	
96	*JAG1*	He	Complete deletion of the gene	Alagille syndrome	
97	*JAG1*	He	**c.1899dupT:p.Glu634_Ser635delinsTer**	Alagille syndrome	
98	*JAG1*	He	c.551G>A:p.Arg184His	Alagille syndrome	
99	*JAG1*	He	c.1977G>A:p.Trp659Ter	Alagille syndrome	
100	*JAG1*	He	**c.286T>C:p.Ser96Pro**	Alagille syndrome	
101	*JAG1*	He	c.2321G>A:p.Trp774Ter	Alagille syndrome	ABCB11 : p.Asp843His (He)
102	*JAG1*	He	c.269_270delinsG :p.Gly90Valfs	Alagille syndrome	
103	*JAG1*	He	**c.635G>C:p.Cys212Ser**	Alagille syndrome	
104	*JAG1*	He	**c.446_449del:p.Asp149Valfs*11**	Alagille syndrome	
105	*JAG1*	He	**c.886G>T:p.Asp296Tyr**	Alagille syndrome	
106	*JAG1*	He	**c.764A>G:p.Tyr255Cys**	Alagille syndrome	
107	*JAG1*	He	c.2230C>T:p.Arg744Ter	Alagille syndrome	
108	*JAG1*	He	**c.107A>G:p.Glu36Gly** (LP)/ **c.185G>C:p.Gly62Ala** (VOUS)	Alagille syndrome	
109	*JAG1*	He	**c.409dupG:p.Glu137Glyfs*160**	Alagille syndrome	
110	*JAG1*	He	Complete deletion of the gene	Alagille syndrome	
111	*JAG1*	He	c.1485_1486del :p.Cys496PhefsTer9	Alagille syndrome	
112	*JAG1*	He	**c.92_98del:p.Ala31Valfs*13**	Alagille syndrome	
113	*JAG1*	He	**c.1264dupG:p.Val422Glyfs*7**	Alagille syndrome	
114	*JAG1*	He	**c.640C>T:p.Gln214Ter**	Alagille syndrome	ABCB4: Ala934Thr He (father)
115	*JAG1*	He	**c.2572+1G>A: p.(?)**	Alagille syndrome	
116	*JAG1*	He	**c.1499delG:p.Gly500Valfs*64**	Alagille syndrome	
117	*JAG1*	He	c.2732G>T:p.Cys911Phe	Alagille syndrome	

118	*JAG1*	He	**c.2840dupA:p.Cys948Valfs*4**	Alagille syndrome	
119	*JAG1*	He	**c.2828C>T:p.Pro943Leu**	Alagille syndrome	
120	*JAG1*	He	**c.82-1G>A: p.(?)**	Alagille syndrome	
121	*JAG1*	He	c.439C>T:p.Gln147Ter	Alagille syndrome	
122	*VPS33B*	Ho	**c.989delA:p .(?)**	ARC syndrome	
123	*VPS33B*	Ho	**c.1148T>A:p.Ile383Asn**	ARC syndrome	
124	*VPS33B*	CHe	**c.1246C>T; p.Arg416Ter** **c.1714T>G:p.Phe572Val**	ARC syndrome	
125	*VPS33B*	Ho	**c.1148T>A:p.Ile383Asn**	ARC syndrome	
126	*VPS33B*	Ho	**c.1160T>G:p.Ile387Arg**	ARC syndrome	
127	*ATP8B1*	Ho	c.1982T>C:p.Ile661Thr	BRIC1	
128	*ABCB11*	CHe	c.1460G>A:p.Arg487His c.2495G>A:p.Arg832His	BRIC2	
129	*ABCB11*	He	c.2095T>C:p.Ser699Pro	BRIC2	
130	*ABCB11*	CHe	**c.23G>A:p.Arg8Gln**; c.3628A>C:p.Thr1210Pro	BRIC2	
131	*AKR1D1*	Ho	c.242A>T:p.Asp81Val	Bile acid synthesis defect	
132	*UGT1A1*	Ho	c.720-730del :p.Arg240SerfsTer14	Criggler Najjar	
133	*CFTR*	CHe	c.349C>T:p.Arg117Cys c.2052delA: p.Lys684AsnfsTer38	Cystic fibrosis	
134	*CFTR*	CHe	c.3909C>G:p.Asn1303Lys c.4054C>T:p.Gln1352Ter	Cystic fibrosis	
135	*CFTR*	Ho	c.1520_1522del; p.Phe508del	Cystic fibrosis	
136	*ABCC2*	Ho	**c.974C>G:p.Ser325Ter**	Dubin-Johnson syndrome	
137	*ABCC2*	Ho	**c.1609_1611del:p.Leu537del**	Dubin-Johnson syndrome	
138	*ABCC2*	2 He	**c.2358delC:p.Leu788CysfsTer8** **c.2901C>A:p.Tyr967Ter**	Dubin-Johnson syndrome	
139	*ABCC2*	CHe	**c.1291delC: p.Leu431SerfsTer4** **c.3918_3919insGTGCG:p.Tyr1309CysfsTer45**	Dubin-Johnson syndrome	ABCB11:p.Gln466Lys (He)
140	*ABCC2*	Ho	c.2362_2363del:p.788_788del	Dubin-Johnson syndrome	ABCB11:p.Asn591Ser (He)
141	*ABCC2*	Ho	**c.4120C>T:p.Arg1374Ter**	Dubin-Johnson syndrome	
142	*ABCC2*	CHe	**c.298C>T:p.Arg100Ter** **c.2997G>A:p.Trp999Ter**	Dubin-Johnson syndrome	
143	*ABCC2*	Ho	c.3196C>T:p.Arg1066Ter	Dubin-Johnson syndrome	CFTR:p.Leu977Phe (He)
144	*ABCC2*	CHe	**c.1963C>T:p.Arg655Ter** **c.3928C>T:p.Arg1310Ter**	Dubin-Johnson syndrome	
145	*ABCC2*	CHe	**c.2388delA:p.Lys797AsnfsTer14** **c.4438C>T:p.Gln1480Ter**	Dubin-Johnson syndrome	
146	*ABCC2*	CHe	c.1216dupT:p.Asp1073Ter **c.delexon26_29:p.(?)**	Dubin-Johnson syndrome	ABCB4:p.Arg47Gln (He)
147	*ABCC2*	CHe	c.974C>G:p.Ser325Ter **c.4117C>T:p.Leu1373Phe**	Dubin-Johnson syndrome	
148	*ABCC2*	Ho	**c.2108A>G:p.Tyr703Cys**	Dubin-Johnson syndrome	
149	*ABCC2*	Ho	c.1216dupT:p.Asp1073Ter	Dubin-Johnson syndrome	
150	*ABCC2*	CHe	**c.4210_4212del:p.1404_1404del** **c.3258+2T>G:p.(?)**	Dubin-Johnson syndrome	
151	*ABCC2*	CHe	c.1216dupT:p.Asp1073Ter c.3196C>T:p.Arg1066Ter	Dubin-Johnson syndrome	
152	*ABCC2*	Ho	c.974C>G:p.Ser325Ter	Dubin-Johnson syndrome	
153	*UGT1A1*	2he	c.1091C>T:p.Pro364Leu c.211G>A:p.Gly71Arg	Gilbert syndrome	
154	*DCDC2*	Ho	c.256delT:p.Tyr86fs	Neonatal sclerosing cholangitis	

155	*DCDC2*	CHe	**c.184G>T:p.Val62Phe**<br>**c.del-exon1:p.(?)**	Neonatal sclerosing cholangitis	Confirmed by qPCR
156	*DCDC2*	CHe	**c.193dupA:p.Ile65Asnfs*50**<br>**c.del-exon5:p.(?)**	Neonatal sclerosing cholangitis	Confirmed by qPCR
157	*DCDC2*	ho	**c.122T>A:p.Val41Glu**	Neonatal sclerosing cholangitis	
158	*DCDC2*	Ho	**c.293+96_294del:p.Asn98LysfsTer2**	Neonatal sclerosing cholangitis	
159	*DCDC2*	Ho	**c.122T>A:p.Val41Glu**	Neonatal sclerosing cholangitis	
160	*DCDC2*	CHe	**c.543C>A:p.Ser181Arg**<br>**c.715T>G:p.Ser239Ala**	Neonatal sclerosing cholangitis	
161	*DCDC2*	Ho<br>Ho	**c.276A>T; p.Glu92Asp**<br>**c.229C>G;p.Leu77Val**	Neonatal sclerosing cholangitis	
162	*DCDC2*	Ho	**c.122T>A:p.Val41Glu**	Neonatal sclerosing cholangitis	
163	*ATP8B1*	CHe	c.2600G>A :p.Arg867His<br>c.782-12T>G: p(?)	PFIC1	CFTR:p.Arg117His (He)
164	*ABCB11*	Ho	c.1409G>A:p.Arg470Gln	PFIC2	
165	*ABCB11*	Ho	c.1062T>A :p.Tyr354Ter	PFIC2	
166	*ABCB11*	Ho	c.3766-2A>T:p.(?)	PFIC2	
167	*ABCB11*	Ho	c.1827_1828insCA:p.Ile610GlnfsTer45	PFIC2	
168	*ABCB11*	Ho	c.677C>T:p.Ser226Leu	PFIC2	
169	*ABCB11*	CHe	c.3491delT:p.Val1164GlyfsTer7<br>c.890A>G :p.Glu297Gly	PFIC2	
170	*ABCB11*	Ho	c.2125G>A:p.Glu709Lys	PFIC2	
171	*ABCB11*	CHe	c.1159C>T:p.Arg387Cy<br>c.2594C>T:p.Ala865Val	PFIC2	
172	*ABCB11*	CHe	c.2125G>A:p.Glu709Lys<br>c.3148C>T:p.Arg1050Cys	PFIC2	
173	*ABCB11*	Ho	c.3637G>A:p.Gly1213Arg	PFIC2	
174	*ABCB11*	CHe	c.2931delA:p.Ala978ProfsTer29<br>c.T1062A:p.Tyr354Ter	PFIC2	
175	*ABCB11*	CHe	c.1459C>T:p.Arg487Cys<br>c.1392dupA:p.Leu465ThrfsTer9	PFIC2	
176	*ABCB11*	Ho	c.3892G>A:p.Gly1298Arg	PFIC2	
177	*ABCB11*	Ho	c.2494C>T:p.Arg832Cys	PFIC2	
178	*ABCB11*	CHe	c.2594C>T:p.Ala865Val<br>c.1159C>T:p.Arg387Cys	PFIC2	
179	*ABCB11*	CHe	c.2177_218+1delins:p.(?)<br>c.dup_exon14-28:p(?)	PFIC2	Confirmed by qPCR
180	*ABCB11*	Ho	c.1827_1828insCA:p.Ile610GlnfsTer45	PFIC2	
181	*ABCB11*	Ho	c.677C>T:p.Ser226Leu	PFIC2	
182	*ABCB11*	Ho	c. 3400C>T:p.Gln1134Ter	PFIC2	
183	*ABCB4*	CHe	c.2860G>A:p.Gly954Ser<br>**c.760G>A:p.Ala254Thr**	PFIC3	
184	*ABCB4*	CHe	c.139C>T :p.Arg47Ter<br>c :1712delT :p.Val571AspfsTer16	PFIC3	
185	*ABCB4*	Ho	**c.2906G>A:p.Arg969His**	PFIC3	
186	*ABCB4*	CHe	**c.2301dupT:p.Thr768TyrfsTer26**<br>c.2800G>A:p.Ala934Thr	PFIC3	
187	*ABCB4*	CHe	c.430C>T:p.Arg144Ter<br>c.1769G>A p.Arg590Gln* (10,11)	PFIC3	
188	*ABCB4*	Ho	c.79A>G:p.Ser27Gly	PFIC3	
189	*ABCB4*	CHe	c.2800G>A:p.Ala934Thr<br>**c.2932T>C: p.Ser978Pro**	PFIC3	
190	*ABCB4*	CHe	**c.1778C>A ; p.Thr593Lys**<br>**c.3409C>T ; p.Arg1137Trp**	PFIC3	
191	*ABCB4*	Ho	**c.3650T>C:p.Leu1217Pro**	PFIC3	
192	*ABCB4*	CHe	**c.2704A>G:p.Asn902Asp**<br>c.delexon9_10:p.(?)	PFIC3	
193	*ABCB4*	Ho	**c.1894-1G>T:p.(?)**	PFIC3	

194	*ABCB4*	CHe	c.2132T>C:p.Phe711Ser<br>c.delexon10_13:p.(?)	PFIC3	ABCB11 : c.1139delT:p.Leu380fs (He)
195	*ABCB4*	Ho	**c.425delC:p.(?)**	PFIC3	
196	*ABCB4*	Ho	**c.1529A>C:p.Asn510Thr**	PFIC3	
197	*ABCB4*	Ho	**c.2545T>C:p.Ser849Pro**	PFIC3	
198	*ABCB4*	CHe	c.2800G>A:p.Ala934Thr<br>c.1348_1353del:p.450_451del	PFIC3	
199	*ABCB4*	Ho	c.101C>T :p.Thr34Met	PFIC3	
200	*TJP2*	Ho	**c.1594G>C p.Gly532Arg**	PFIC4	
201	*TJP2*	CHe	**c.2556delC :p.Leu853PhefsTer8**<br>c.delexon1_16:p.(?)	PFIC4	
202	*TJP2*	CHe	**c.3283C>T:p.Gln1095Ter**<br>c.dupexon5 _17: p.(?)	PFIC4	
203	*TJP2*	Ho	**c.784G>T:p.Glu262Ter**	PFIC4	
204	*TJP2*	Ho	**c.C847T:p.Glu283Ter**	PFIC4	
205	*TJP2*	CHe	**c.1249G>C:p.Glu417Gln**<br>**c.1383_1386del:p.461_462del**	PFIC4	
206	*TJP2*	Ho	**c.953-1G>C:p.(?)**	PFIC4	
207	*TJP2*	Ho	**c.1894C>T:p.Arg632Ter**	PFIC4	
208	*NR1H4*	CHe	c.526C>T:p.Arg176Ter<br>**c.1180+2T>C:p.(?)**	PFIC5	
209	*MYO5B*	CHe	**c.731T>A:p.Leu244Ter**<br>**c.1207G>A:p.Ala403Thr**	Myosin 5b deficiency related cholestasis (in absence of MVID)	
210	*MYO5B*	Ho	c.356A>G:p.Tyr119Cys	Myosin 5b deficiency related cholestasis (in absence of MVID)	
211	*MYO5B*	CHe	c.1135C>T:p.Arg379Cys (mother)<br>c.1906-2A>G:p.(?) (mother)<br>c.2470C>T:p.Arg824Cys (father)	Myosin 5b deficiency related cholestasis (in absence of MVID)	
212	*MYO5B*	CHe	c.274C>T:p.Arg92Cys<br>**c.283G>A:p.Glu95Lys**	Myosin 5b deficiency related cholestasis (in absence of MVID)	
213	*MYO5B*	CHe	c.2395C>T:p.Arg799Trp<br>c.delexon2_27:p.(?)	Myosin 5b deficiency related cholestasis (in absence of MVID)	
214	*MYO5B*	CHe	**c.1675T>C:p.Tyr559His**<br>**c.860A>G:p.Tyr287Cys**	Myosin 5b deficiency related cholestasis (in absence of MVID)	
215	*MYO5B*	CHe	c.274C>T:p.Arg92Cys<br>c.2395C>T:p.Arg799Trp	Myosin 5b deficiency related cholestasis (in absence of MVID)	
216	*MYO5B*	3He	c.1925T>C:p.Leu642Pro<br>c.1499T>C:p.Ile500Thr	Myosin 5b deficiency related cholestasis (in absence of MVID)	
217	*MYO5B*	Ho	c.2470C>T : p.Arg824Cys	Myosin 5b deficiency related cholestasis (in absence of MVID)	
218	*MYO5B*	CHe<br>CHe	***MYO5B*:c.5323delA:p.Ile1775PhefsTer2**<br>*MYO5B*:c.274C>T:p.Arg92Cys	Myosin 5b deficiency related	

			***ATP8B1*:c.163C>T :p.Arg55Trp**<br>*ATP8B1*:c.134A>C:p.Asn45Thr	cholestasis (in absence of MVID) and/or PFIC1	
219	*CYP27A1*	Ho	c.446+1G>A :p(?)	Cerebrotendinous xanthomatosis	
220	*ACOX2*	Ho	**c.461_464del:p.Thr154fs**	Primary bile acid synthesis defect	
221	*ACOX2*	Ho	**c.73G>A:p.Glu25Lys**	Primary bile acid synthesis defect	
222	*BAAT*	Ho	**c.1205T>A:p.Ile402Asn**	Primary bile acid synthesis defect (conjugation defect)	
223	*SLCO1B1/ SLCO1B3*	Ho	Complete deletion of both genes	Rotor syndrome	CGHArray checked
224	*SCYL1*	CHe	**c.2352_2353insGA:p.Arg784fs**<br>**c.1386+1G>A:p(?)**	Spinocerebellar ataxia	
225	*SCYL1*	Ho	**c.129_130del:p.Ser44HisfsTer5**	Spinocerebellar ataxia	
226	*SCYL1*	Ho	**c.1346_1347del:p.Thr449SerfsTer13**	Spinocerebellar ataxia	
227	*TTC37*	2 He	**c.4514T>C:p.Leu1505Ser**<br>**c.3015-1G>A.p(?)**	Tricho-hepato-enteric syndrome	
228	*ATP7B*	CHe	c.Exon 1 deletion<br>**c.2495A>C:p.Lys832Thr**	Wilson disease	
229	*ATP7B*	Ho	c.3008C>T :p.Ala1003Val	Wilson disease	
230	*ATP7B*	CHe	c.3809A>G:p.Asn1270Ser<br>**c.366_367insX:p.Ala123delins**	Wilson disease	

Ho, homozygous, He, heterozygous; CH compound heterozygous, MVID: microvillous inclusion disease. *The pathogenicity of this variant is supported by previous publications (10,11). Previously unreported variants according to ClinVar® and Varsome® are indicated in bold.

Table S2. Main characteristics of 2 patients with PFIC3 and cholangiopathy mimicking neonatal sclerosing cholangitis.

	**Patient 188**	**Additionnal patient (unreported in the study)**
**First diagnosis**	Neonatal sclerosing cholangitis	Neonatal sclerosing cholangitis
**Liver Histology**	- Peribiliary fibrosis<br>- Few missing intralobular bile ducts<br>- Ductular proliferation<br>- Septal fibrosis	- Cholangitis<br>- Ductopenia<br>- Ductular proliferation<br>- Septal fibrosis
**Cholangiogram**	Irregular intrahepatic bile ducts (MRI-cholangiography)	Irregular intrahepatic bile ducts (transhepatic cholescystography)
**Genotype ABBC4**	c.79A>G:p.Ser27Gly (Ho)	c.2800G>A:p.Ala934Thr (CHe)<br>c.1348_1353del:p.450_451del (CHe)
**Canalicular MDR3 immunostaining**	Negative	Faint
**Biliary phospholipids (% of total biliary lipids, N: 19-24%)**	Not available	14%
**Evolution**	- Cirrhosis<br>- LT at 10 years old	- Cirrhosis<br>- Normal LFT with UDCA treatment at 11 years old

UDCA : ursodeoxycholic acid, LFT : liver function tests, LT : liver transplantation.

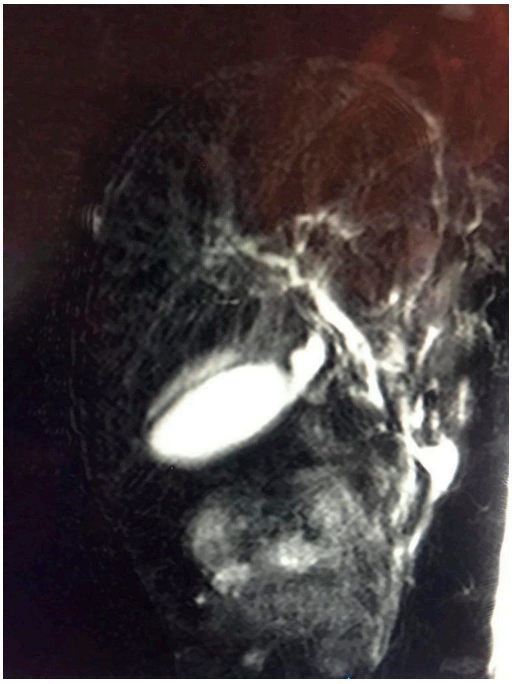

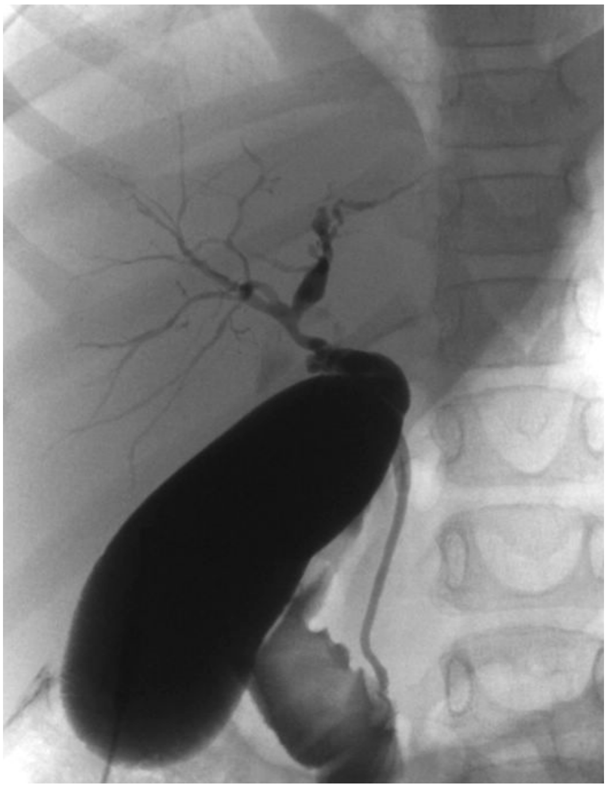

a **b**

Figure S2. a: MRI-cholangiography in patient 188 with MDR3 deficiency (PFIC3). Cholangiogram showed changes in the intrahepatic bile ducts mimicking neonatal sclerosing cholangitis. **b**: Transhepatic cholecystography in an additional PFIC3 patient not included in the study. Cholangiogram showed changes in the intrahepatic bile ducts mimicking neonatal sclerosing cholangitis. These changes consisted of irregularities of the duct wall, filling defects, irregular dilation and rarefaction of small biliary branches.
